# Editorial: Complexity and emergence in brain network analyses

**DOI:** 10.3389/fncom.2015.00065

**Published:** 2015-06-02

**Authors:** Qawi K. Telesford, Sean L. Simpson, Eric D. Kolaczyk

**Affiliations:** ^1^Complex Systems Group, Department of Bioengineering, University of PennsylvaniaPhiladelphia, PA, USA; ^2^Laboratory for Complex Brain Networks, Division of Public Health Sciences, Wake Forest University School of MedicineWinston-Salem, NC, USA; ^3^Department of Mathematics and Statistics, Boston UniversityBoston, MA, USA

**Keywords:** brain networks, complex systems, network dynamics, network analysis, group analysis, functional imaging

Network science has become an invaluable tool for neuroimaging analysis and has advanced our understanding of the brain's complex structural and functional topology. Network science, an interdisciplinary offshoot of graph theory, conceives of the brain as a system of nodes, representing brain regions or voxels, and edges, representing the structural or functional connection between these regions or voxels. Such a model is considered appealing in neuroscience as it describes a system with various interacting regions that produces complex behaviors. This conception of the brain has revealed that the brain exhibits “small-world” properties, namely that brain networks are systems with regional specialization and efficient global information transfer. More importantly, studies in brain networks have furthered our understanding of diseases and disorders affecting the brain.

Assessing group differences in representative samples of brain networks has become increasingly important, yet appropriate methodological developments have remained relatively sparse. Additionally, there is increased interest in understanding dynamic changes, or how a network changes over time. Currently, most network analysis tools are designed to analyze a static representation, thus changes that occur over time are often overlooked or ignored. As the field continues to grow, it is becoming increasingly important to develop methods for understanding the complexity of these data. This research topic features selected contributions that explores group analysis and dynamics in brain network studies including methodological and practical applications employing various imaging modalities. This snapshot of the field represents the growing interest to expand and validate new methods for use in the field of brain network neuroscience.

“Defining nodes in complex brain networks” is a review article discussing the methodological choices for defining nodes, particularly how the choice of atlas-based or voxel-based methods affects the computed network (Stanley et al., 2013).

“Routing in the brain” is a commentary on network routing focusing on how current methods and models may not be adequate for brain networks. Further exploration of the packet switching network model may be more realistic and appropriate for the brain (Graham, 2014).

“A multimodal approach for determining brain networks by jointly modeling functional and structural connectivity” is a methods article introducing a Bayesian model for estimating functional networks, from functional magnetic resonance imaging (fMRI) data, leveraging complementary structural diffusion tensor imaging (DTI) data (Xue et al., 2015).

“A permutation testing framework to compare groups of brain networks” is a methods article describing a permutation testing framework to statistically compare groups of functional brain networks while incorporating topological features inherent in each individual network (Simpson et al., 2013).

“Assessing dynamics, spatial scale, and uncertainty in task-related brain network analyses” is a methods article describing a statistical approach for computing uncertainty in static and dynamic functional networks and aggregating network measures in task-related electrocorticography (ECoG) data (Stephen et al., 2014).

“Hierarchical vector auto-regressive models and their applications to multi-subject effective connectivity” is a methods article introducing a generalization of the vector auto-regressive (VAR) model for comparing effective connectivity between experimental conditions while accounting for between-subject heterogeneity (Gorrostieta et al., 2013).

“Statistical network analysis for functional MRI: summary networks and group comparisons” is a review article discussing the construction of summary networks and how to test for topological differences in groups of networks when these groups also exhibit significant differences in density (Ginestet et al., 2014).

“Detecting functional connectivity change points for single-subject fMRI data” is a methods article that extends Dynamic Connectivity Regression (DCR), a data-driven technique which detects change points in functional connectivity between brain regions to determine state changes over the course of an experimental task, by introducing a novel algorithm aimed at increasing the estimation of networks for individual subject data (Cribben et al., 2013).

“The Laplacian spectrum of neural networks” is an original research article examining the use of the Laplacian spectrum in anatomic networks of the macaque, cat and Caenorhabditis elegans. This method describes network structure at a systems level, assessing systemic infrastructural properties of the entire network as opposed to properties of specific nodes or connections (De Lange et al., 2014).

“Quantifying network properties in multi-electrode recordings: spatiotemporal characterization and inter-trial variation of evoked gamma oscillations in mouse somatosensory cortex *in vitro*” is an original research article on network dynamics in multi-electrode array data focusing on changes in the functional connectivity of evoked gamma oscillations in cortical circuits (Carmeli et al., 2013).

## Conflict of interest statement

The authors declare that the research was conducted in the absence of any commercial or financial relationships that could be construed as a potential conflict of interest.
